# Bacteremia and Mortality among Patients with Nontunneled and Tunneled Catheters for Hemodialysis

**DOI:** 10.1155/2024/3292667

**Published:** 2024-02-06

**Authors:** Carla Santos De Lima, Flora Braga Vaz, Rodrigo Peixoto Campos

**Affiliations:** ^1^Postgraduate Program in Medical Sciences at the Faculty of Medicine, FAMED, Federal University of Alagoas-UFAL, Maceió, Brazil; ^2^Ribamar Vaz Institute of Nephrology, Santa Casa de Misericórdia of Maceió, Maceió, Brazil

## Abstract

**Introduction:**

Central venous catheters for hemodialysis (HD) can be nontunneled catheters (NTC) or tunneled catheters (TC). Bacteremia and dysfunction are complications that can impact morbidity and mortality. We decided to compare the rates of bacteremia and dysfunction between NTC and TC and patient survival 90 days after catheter insertion.

**Methods:**

Retrospective cohort to evaluate catheters inserted between January 2011 and December 2020 in a tertiary hospital. Catheters in patients with end-stage chronic kidney disease were included. Patients with acute kidney injury, catheters that lasted less than three HD sessions, and patients who died within one week after insertion were excluded. Bacteremia and dysfunction rates, bacteremia-free survival, and dysfunction-free survival were investigated. Multivariable analysis was performed using a Cox proportional hazards regression model for patient survival at 90 days.

**Results:**

670 catheters were analyzed in 287 patients, 422 NTC (63%), and 248 TC (37%). The rates of confirmed bacteremia per 1,000 catheter-days were 1.19 for NTC and 0.20 for TC (*p* < 0.0001). The confirmed or possible bacteremia rates were 2.27 and 0.37 per 1,000 catheter-days for NTC and TC, respectively (*p* < 0.0001). The dysfunction rates were 3.96 and 0.86 for NTC and TC, respectively (*p* < 0.0001). Patient survival at 90 days was higher in the TC group than the NTC group (96.8% vs. 89.1%; *p* < 0.0001).

**Conclusion:**

We found lower rates of bacteremia and dysfunction for TC and demonstrated that using NTC affects patient mortality.

## 1. Introduction

Chronic kidney disease (CKD) is a clinical syndrome based on the presence of kidney damage or reduced kidney function (glomerular filtration rate (GFR) < 60 ml/min/1.73 m^2^) for three months or more [1]. When GFR is less than 15 ml/min/1.73 m^2^, kidney function is not capable of maintaining life for a long time [2], and renal replacement therapy (RRT) such as hemodialysis (HD) or kidney transplantation is necessary to prolong life and reverse uremic symptoms [3]. For HD to be performed, patients must have vascular access for hemodialysis. Central venous catheters (CVC) are indicated in cases of emergency hemodialysis or, in chronic hemodialysis, in cases where it is not possible to perform an arteriovenous fistula [4] or where it is dysfunctional [5].

Tunneled cuffed catheters for hemodialysis were developed as a technological breakthrough to anchorage it subcutaneously and to reduce infections as used in other circumstances, such as peritoneal dialysis, chemotherapy, and parenteral nutrition. In Brazil, according to the Brazilian Dialysis Census, 23.6% of patients with hemodialysis (HD) use central venous catheters (catheters), 9.2% of nontunneled catheters (NTC), and 14.4% of tunneled catheters (TC) [6]. The use of a catheter for HD access is associated with an increase in all-cause mortality compared to the use of arteriovenous fistula (AVF) [7], and some complications may be related to the presence of a catheter, including infections [5] and dysfunction [8]. Catheter-related infections are responsible for significant rates of hospitalization and mortality [9], especially those that progress to bacteremia [10], while dysfunction can imply a reduction in HD effectiveness [8]. Interestingly, previous studies report that TC has lower bacteremia rates than NTC [11, 12].

Despite continuing progress in hemodialysis therapy, the mortality rate is unacceptably high in catheter patients [13]. In addition, other factors are associated with the high prevalence of mortality, especially cardiovascular disease [14], anemia, inflammation, hypoalbuminemia [15], and low-dose hemodialysis, quantified by Kt/V [16].

Furthermore, we evaluated bacteremia and dysfunction rates for NTC and TC in a large single-center retrospective cohort and risk factors for 90-day survival in patients who used catheters as vascular access to HD.

## 2. Methodology

### 2.1. Study Design

This retrospective cohort study evaluated catheters inserted in end-stage renal disease patients from January 1, 2011, to December 31, 2020, in a tertiary hospital in Northeast Brazil. Patients undergoing hemodialysis due to acute kidney injury, catheters lasting less than 3 (three) hemodialysis sessions, death within one week after insertion, or transfer to another hemodialysis service within one week after hemodialysis catheter insertion were excluded. The NTCs were inserted by a nephrologist in a specific room for minor surgical procedures, without ultrasound-guided, while the TCs were inserted by a vascular surgeon guided by fluoroscopy. The catheter insertion site and the catheter model were defined by the physician who would perform the implant. The nontunneled catheter model used was Arrow® (Teleflex). Tunneled catheter models were Split Cath® (Medcomp), Palindrome™ (Medtronic), and Equistream™ (BD). All patients were in conventional 4-hour hemodialysis sessions with low-flux dialyzers. Blood flow rates were prescribed individually to reach a single-pool Kt/V of 1.3. In all dialysis sessions, the exit site and the subcutaneous tunnel were evaluated by the nursing team according to the institution protocol, and the dressing material was changed with sterile gauze after cleaning with 2% alcoholic chlorhexidine. The study was approved by the local Medical Ethics Committee (No. 5,079,821, of November 4, 2021).

### 2.2. Data Collection

All medical data were collected from the electronic medical record. Clinical and laboratory data such as age, diagnosis of diabetes mellitus, serum levels of hemoglobin, and albumin were collected at catheter insertion. The Kt/V was calculated after catheter insertion. All information about the catheter was also included: type of catheter used (nontunneled or tunneled), insertion site, catheter survival, reasons for catheter removal, and occurrence of death. All catheters were followed from the time of insertion until removal.

### 2.3. Definitions and Outcomes

Catheter-related bacteremia was diagnosed as confirmed or possible confirmed as follows:

*Confirmed bacteremia* was defined as the presence of the same organism from a semiquantitative culture of the catheter tip and from a peripheral or catheter blood sample in a symptomatic patient with no other apparent source of infection [17].

*Possible bacteremia* was defined as the presence of symptoms related to bacteremia that occurred without signs and symptoms of infection related to other sites and with negative cultures (blood culture or catheter tip culture) but with clinical improvement and defervescence with catheter removal and the initiation of antibiotic therapy [17].

Although the exit site and tunnel infection were not part of the primary outcome, these outcomes were evaluated and diagnosed according to the KDOQI criteria [8]. If patients with exit site or tunnel infection had systemic symptoms, they were characterized as bacteremia. Following the institutional protocol for possible catheter-related bacteremia, two blood samples (peripheral, from the hemodialysis circuit or catheter) were collected in blood culture bottles (Bactec 9240, Becton Dickinson). Until 2018, catheter-tip segment cultures were performed. All catheters with possible or confirmed infection are routinely removed at the institution.

*Catheter dysfunction* was confirmed when the catheter did not provide blood flow greater than 200 ml/min for more than 2 (two) hemodialysis sessions. The catheter was removed, and a new catheter was inserted.

The *primary outcome* was to assess the rates of possible and confirmed bacteremia per 1,000 catheter-days and bacteremia-free survival among nontunneled and tunneled catheters. The dysfunction rate and dysfunction-free survival were also analyzed. Catheters with bacteremia and dysfunction simultaneously were defined as catheter-related bacteremia.

The *secondary outcome* was to analyze patient survival in the first 90 days of catheter use. Variables such as age, diabetes mellitus, type of catheter, insertion site, bacteremia, dysfunction, hemoglobin, albumin, and Kt/V were included as predictors.

### 2.4. Statistical Analysis

Statistical analysis was performed using the IBM SPSS® version 20.0 program. Data were presented using the mean ± standard deviation, median (1^st^ and 3^rd^ quartiles), and percentage rates. Normally distributed variables were compared using Student's *t*-test, and nonnormally distributed variables were compared using the Mann–Whitney *U* test. The chi-square test was used to compare categorical variables. Bacteremia and dysfunction rates (events per 1,000 catheter-days) were compared using the log-rank test. The Kaplan–Meier method and the log-rank test were used to analyze bacteremia-free and dysfunction-free survival. All patients were censored in the survival analysis if the arteriovenous access was mature, if they were transferred to peritoneal dialysis, if they underwent kidney transplantation, or if they were lost to follow-up. For the analysis of patient survival at 90 days, an unadjusted and adjusted analysis was performed using the Cox proportional hazards regression model and presented as the hazard ratio (HR) and 95% confidence intervals (95% CI). For adjusted analysis, we included age, diabetes, catheter type, vein location, presence of confirmed bacteremia and dysfunction, hemoglobin, and albumin. The *p* value was considered significant at *p* < 0.05.

## 3. Results

During the period, 1,914 catheters for hemodialysis were inserted. After applying the inclusion and exclusion criteria, a total of 670 catheters inserted in 287 patients were evaluated; 422 (63%) were nontunneled, and 248 (37%) were tunneled. Clinical and catheter characteristics are shown in Table 1. The median serum albumin was 3.5 g/dl (3.1–3.9) in NTC and 3.7 g/dl (3.4–4) in TC (*p* < 0.0001). The median Kt/V was 1.25 (1.1–1.5) in NTC and 1.3 (1.1–1.5) for TC (*p*=0.005). The median time of TC was longer than that of NTC (185 vs. 37 days) (*p* < 0.0001). The right internal jugular vein was both groups' most common insertion site.

### 3.1. Bacteremia and Dysfunction

**Figure 1:**
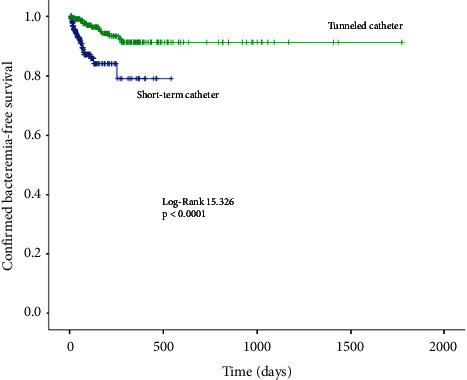
Kaplan–Meier curve for confirmed bacteremia-free survival.

**Figure 2:**
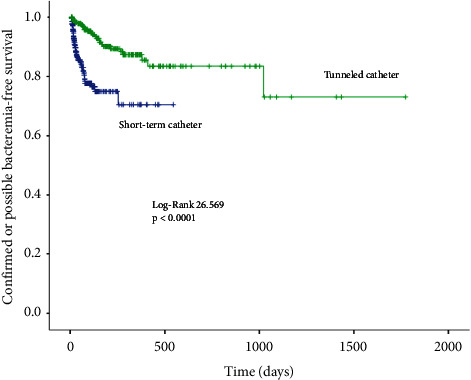
Kaplan–Meier curve for confirmed or possible bacteremia-free survival.

A total of 92,008 days were analyzed (27,751 days for NTC and 64,257 days for TC).  Table 2 displays the event rate between nontunneled and tunneled catheters. Confirmed bacteremia occurred in 33 nontunneled catheters (7.8%) and 13 tunneled catheters (5.2%) (*p*=0.203). However, the confirmed bacteremia rate per 1,000 catheter-days was 1.19 for NTC and 0.2 for TC (*p* < 0.0001) (Figure 1). The incidence of confirmed or possible bacteremia occurred in 63 (14.9%) NTC and 24 (9.7%) TC (*p*=0.051). The confirmed or possible bacteremia rate was 2.27 per 1,000 catheter-days for NTC and 0.37 for tunneled catheters (*p* < 0.0001) (Figure 2).

**Figure 3:**
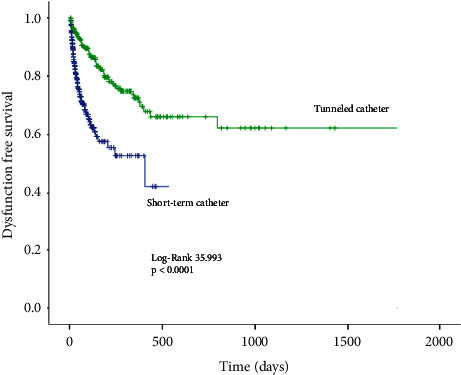
Kaplan–Meier curve for dysfunction-free survival.

**Figure 4:**
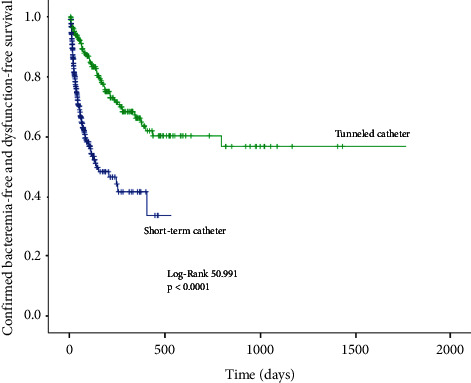
Kaplan–Meier curve for confirmed bacteremia-free and dysfunction-free survival.

**Figure 5:**
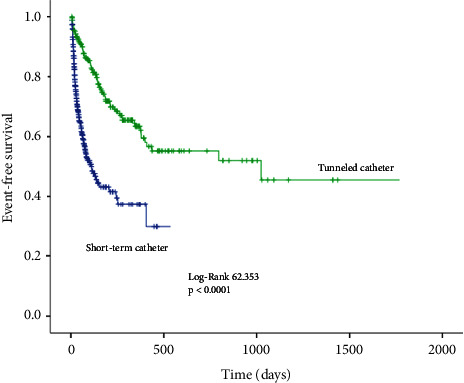
Kaplan–Meier curve for event-free survival.

Dysfunction occurred in 110 (26.01%) nontunneled catheters and 55 (22.2%) tunneled catheters (*p*=0.259). The dysfunction rate was 3.96 per 1000 catheter-days for NTC and 0.86 per 1000 catheter-days for TC (*p* < 0.0001) (Figure 3). Confirmed bacteremia or dysfunction was observed in 143 (33.9%) NTC and 68 (27.4%) TC (*p*=0.082). Confirmed bacteremia or dysfunction was 5.15 per 1,000 catheter-days for NTC and 1.06 for tunneled catheters (*p* < 0.0001) (Figure 4). The occurrence of any of the above events (confirmed or possible bacteremia or dysfunction) had an incidence of 41% for NTC and 31.8% for TC (*p*=0.018), representing 6.23 and 1.23 per 1,000 catheter-days, respectively (*p* < 0.0001) (Figure 5).

### 3.2. Bacteriological Profile

The microbiological profile of cultures with bacteremia is shown in Table 3.

### 3.3. Survival

Ninety-day survival was better for TC versus NTC (96.8% vs. 89.1%, respectively; *p* < 0.0001). The Cox regression unadjusted analysis indicated a mortality risk of 3% for each year of life (HR 1.03; 95% CI 1.02–1.05; *p* < 0.001). For the use of the nontunneled catheter, the HR was 3.79 (95% CI: 2.29–6.01; *p* < 0.001). Concerning serum albumin levels, each increase of 1 g/dl reduced the mortality risk by 53% (HR 0.47; 95% CI 0.36–0.62; *p* < 0.001). However, applying the Cox regression adjusted analysis showed a mortality risk of 4% for each year of life (HR 1.04; 95% CI 1.02–1.05; *p* < 0.001). For the use of the nontunneled catheter, the HR was 4.64 (95% CI: 2.76–7.8; *p* < 0.001). Each increase of 1 g/dl in serum albumin levels reduced the mortality risk by 49% (HR 0.51; 95% CI 0.37–0.70; *p* < 0.001). The other variables (diagnosis of diabetes mellitus, jugular vein, dysfunction, bacteremia, hemoglobin, and Kt/V) were not statistically significant in the adjusted analysis of 90-day survival (Table 4).

## 4. Discussion

Central venous catheters are often used as vascular access for hemodialysis. However, device infection is one of the most severe complications [18]. Similarly, catheter dysfunction is a common complication associated with reduced adequacy, increased risk of catheter-related bloodstream infection, and mortality [8]. This study demonstrated higher bacteremia rates and dysfunction in nontunneled catheters for outpatient hemodialysis.

Sahli et al. demonstrated in a study with nontunneled catheters in outpatients an infection rate of 16.6 per 1,000 catheter-days, with a catheter-related bacteremia rate of 10.8 per 1,000 catheter-days [19]. Moran et al. found a bacteremia rate of 0.91 per 1,000 catheter-days [20], while Maki et al. (2011) documented a bacteremia rate of 0.82 per 1,000 catheter-days [21] for tunneled catheters. Martin et al. found a confirmed or possible bacteremia rate for tunneled hemodialysis catheters of 1.28 per 1,000 catheter-days [22]. A previous study in Singapore, a country with a tropical climate similar to Brazil, revealed a bacteremia rate in tunneled HD catheters of 0.75 per 1,000 catheter-days [23]. In our cohort, the incidence of bacteremia (possible or confirmed) was 2.27 per 1,000 catheter-days for nontunneled catheters and 0.37 per 1,000 catheter-days for tunneled catheters.

There is variability between catheter-related bacteremia rates for HD in previous studies. The bacteremia rates found in our study were low for nontunneled and tunneled catheters. A possible explanation for the reduced rates of catheter-associated infection was the follow-up of protocols and care by the nursing team throughout the hemodialysis session, which includes the use of 2% chlorhexidine to clean the exit site of the catheter, in addition to a continuing education program on the prevention of catheter complications. Previous studies have shown reduced bacteremia in patients after establishing a catheter care procedure using 2% chlorhexidine [24]. Furthermore, continuing education for patients and their families and an easy access route for patients and healthcare professionals to get help with catheter problems is the key to maintaining low bacteremia rates [23].

A previous study reported that coagulase-negative Staphylococcus aureus was the pathogen most commonly isolated in nontunneled catheter infections [25]. Similarly, another study noted that Gram-positive microorganisms were responsible for most cases of tunneled catheter infection, and 40 to 81% of infections were caused by *Staphylococcus aureus* [26]. An Indian study indicated high bacteremia rates in tunneled catheter patients (42% at 180 days) with a higher incidence of Gram-negative bacteria growth in blood cultures. This finding was attributed to the low socioeconomic status of patients, poor hygiene, and water contamination in the HD service [27]. In our study, episodes of *Staphylococcus aureus* bacteremia were predominant only in nontunneled catheters. This may be due to the small number of confirmed bacteremia in tunneled catheters.

Regarding dysfunction, a previous study found an overall incidence rate of catheter dysfunction of 10.58 per 1,000 catheter-days, 12.86 per 1,000 catheter-days for nontunneled catheters, and 8.64 per 1,000 catheter-days for tunneled catheters [28]. Our study found a rate of 3.96 per 1,000 catheter-days for nontunneled catheters and 0.86 per 1,000 catheter-days for tunneled catheters. The low rate of dysfunction reported in our study can be explained by the fact that we used as a dysfunction criterion the catheter that does not provide blood flow greater than 200 ml/min. According to Griffiths et al., blood flow rates less than 300 ml/min are often used to define hemodialysis catheter dysfunction [8, 29]. HD patients with catheters are associated with hypoalbuminemia compared to patients using AVF as vascular access. This association is multifactorial, and one of the causes is infection and poor dialysis adequacy [30, 31]. Among the characteristics of the patients analyzed, serum albumin levels and Kt/V showed a difference between patients with nontunneled and tunneled catheters, which was higher in patients with tunneled catheters.

The catheter survival time was higher for TC with statistical significance. A study reported a median catheter survival of 62.5 days for TC [32]. In our cohort, the median duration of TC was 185 days. There was a difference in bacteremia and dysfunction-free survival, which was more significant in tunneled catheters. These findings reflect the lower rates of bacteremia and dysfunction in these catheters, with a lower need for removal.

Regarding patient survival 90 days after catheter insertion, there was an additional mortality risk of 3% for each year of patient life. In fact, in a Korean study, elderly patients with catheters as vascular access for HD had higher mortality than those with AVF [33]. An inverse relationship between serum levels and mortality risk has been demonstrated about albumin. Previous studies have shown that in hemodialysis patients, hypoalbuminemia is a risk factor for death [34]. Furthermore, in our study, the mortality risk was also higher in individuals with nontunneled catheters than those with tunneled catheters. A study in Palestine that compared mortality in patients with AVF and catheter as vascular access for HD also reported higher mortality in the catheter group. In this study, most devices in the catheter group were nontunneled catheters [35]. A similar result was found in a study in Sarajevo, which revealed an increase in mortality rates in patients using nontunneled catheters compared to patients using AVF or tunneled catheters [36].

While other studies have linked bacteremia to increased mortality in hemodialysis patients, our study's multivariable regression model did not find it to be a significant risk factor. This may be due to the low occurrence of confirmed bacteremia and the fact that confirmed bacteremia and dysfunction frequently occurred after 90 days for both catheter types (26.1% for bacteremia and 25.5% for dysfunction). However, our study found that a nontunneled catheter predicted poor survival, even without a relationship with bacteremia, in our regression model. Lower serum albumin levels and lower HD adequacy for nontunneled catheter patients may explain this [37].

Our study has some limitations. Firstly, this is a retrospective analysis. Therefore, there is a lack of information, including causes of death, cardiovascular diseases, or other risk factors that impact patient mortality, such as serum phosphorus levels, potassium, inflammatory markers, and blood volume status. Secondly, as the data were collected from medical records, there may have been underreporting of outcomes, which could explain the low incidence of bacteremia and dysfunction. Another significant limitation is the lack of information about catheter type indication. Probably, the indication by the nephrology team of which catheter type should be inserted was decided based on the vascular surgeon and device availability.

In conclusion, tunneled catheters had lower rates of bacteremia and dysfunction than nontunneled catheters. Additionally, nontunneled catheters influenced mortality in the first 90 days after insertion compared to tunneled catheters. According to this study, it is recommended to choose tunneled catheters instead of nontunneled ones for patients undergoing chronic HD treatment. It is wise to consider switching to a tunneled catheter while waiting for an arteriovenous fistula or arteriovenous graft. However, as this is a retrospective study, further studies are needed.

## Figures and Tables

**Table 1:** Clinical and laboratory characteristics of patients and catheters.

	Nontunneled catheter *N* = 422	Tunneled catheter *N* = 248	*p* value
Age (in years)^a^	54.9 (47.3–64.7)	57.3 (43.9–67.1)	0.629
Diabetes mellitus^b^	93 (22%)	59 (23.8%)	0.601
Hemoglobin (g/dl)^a^	9.1 (7.9–10.7)	9.2 (8.2–10.4)	0.614
Albumin (g/dl)^a^	3.5 (3.1–3.9)	3.7 (3.4–4)	<0.0001
Kt/V^a^	1.25 (1.1–1.5)	1.3 (1.1–1.5)	0.005
Catheter use time (days)^a^	37 (17–80)	185 (75–344)	<0.0001
Insertion site^b^			<0.001
Right jugular	194 (46.0)	78 (31.5)	
Left jugular	89 (21.1)	28 (11.3)	
Right subclavian	21 (5.0)	63 (25.4)	
Left subclavian	12 (2.8)	33 (13.3)	
Right femoral	75 (17.8)	23 (9.3)	
Left femoral	31 (7.3)	23 (9.3)	

^a^Median (1^st^ quartile–3^rd^ quartile); ^b^absolute number (%).

**Table 2:** Event rates between nontunneled and tunneled catheters.

	Nontunneled catheter *N* = 422	Tunneled catheter *N* = 248	*p* value
Confirmed bacteremia			
Incidence *n* (%)	33 (7.8)	13 (5.2)	0.203
1,000 catheter-days	1.19	0.20	<0.0001
Confirmed or possible bacteremia			
Incidence *n* (%)	63 (14.9)	24 (9.7)	0.051
1,000 catheter-days	2.27	0.37	<0.0001
Dysfunction			
Incidence *n* (%)	110 (26.1)	55 (22.2)	0.259
1,000 catheter-days	3.96	0.86	<0.0001
Confirmed bacteremia or dysfunction			
Incidence *n* (%)	143 (33.9)	68 (27.4)	0.082
1,000 catheter-days	5.15	1.06	<0.0001
Confirmed bacteremia or possible bacteremia or dysfunction			
Incidence *n* (%)	173 (41.0)	79 (31.8)	0.018
1,000 catheter-days	6.23	1.23	<0.0001

**Table 3:** Bacteriological profile of catheter cultures with bacteremia.

	Nontunneled catheter *N* = 33	Tunneled catheter *N* = 13
*Staphylococcus aureus*	10	1
*Staphylococcus aureus* (MRSA)	7	1
*Klebsiella pneumoniae*	3	0
*Acinetobacter baumannii*	2	0
Coagulase-negative *Staphylococcus* spp.	2	0
*Klebsiella rhinoscleromatis*	1	0
*Staphylococcus lugdunensis*	1	0
*Staphylococcus epidermidis* (MRSE)	1	2
*Staphylococcus capitis*	1	0
*Staphylococcus haemolyticus*	1	0
*Escherichia coli*	1	0
Beta-hemolytic *Streptococcus pyogenes*	1	0
*Staphylococcus* spp.	1	0
*Pseudomonas aeruginosas*	1	0
*Serratia marcesces*	0	2
*Enterobacter aerogenes*	0	2
*Stenotrophomonas maltophilia*	0	1
*Staphylococcus intermedius*	0	1
*Staphylococcus hominis*	0	1
*Burkholderia cepacia*	0	1
*Staphylococcus epidermidis*	0	1

**Table 4:** Cox proportional hazards regression model for 90-day survival.

	Unadjusted			Adjusted		
	HR	95% CI	*p* value	HR	95% CI	*p* value
Age (each year)	1.03	1.02–1.05	<0.001	1.04	1.02–1.05	<0.001
DM	1.33	0.90–1.96	0.146	1.23	0.83–1.85	0.296
Nontunneled catheter	3.79	2.29–6.01	<0.001	4.64	2.76–7.80	<0.001
JIV versus FV	1.20	0.79–1.84	0.393	1.46	0.95–2.26	0.085
JIV versus SCV	0.78	0.48–1.25	0.299	1.57	0.93–2.67	0.093
Hb (every 1 g/dL)	0.96	0.87–1.05	0.357	0.90	0.99–1.17	0.029
Albumin (every 1 g/dL)	0.47	0.36–0.62	<0.001	0.51	0.37–0.70	<0.001
Kt/V (every 1 unit)	1.00	0.59–1.70	0.988	0.62	0.37–1.04	0.069

HR: hazard ratio; CI: confidence interval; DM: diabetes mellitus; JIV: jugular internal vein; FV: femoral vein; SCV: subclavian vein; Hb: hemoglobin.

## Data Availability

The collected data are not available for consultation as they are contained in the medical record, which is a confidential document, thus preventing their disclosure.
